# A VDAC1-Derived N-Terminal Peptide Inhibits Mutant SOD1-VDAC1 Interactions and Toxicity in the SOD1 Model of ALS

**DOI:** 10.3389/fncel.2019.00346

**Published:** 2019-08-14

**Authors:** Anna Shteinfer-Kuzmine, Shirel Argueti, Rajeev Gupta, Neta Shvil, Salah Abu-Hamad, Yael Gropper, Jan Hoeber, Andrea Magrì, Angela Messina, Elena N. Kozlova, Varda Shoshan-Barmatz, Adrian Israelson

**Affiliations:** ^1^Department of Life Sciences, The National Institute for Biotechnology in the Negev, Ben-Gurion University of the Negev, Beersheba, Israel; ^2^Department of Physiology and Cell Biology, Faculty of Health Sciences, The Zlotowski Center for Neuroscience, Ben-Gurion University of the Negev, Beersheba, Israel; ^3^Department of Neuroscience, Uppsala University, Uppsala, Sweden; ^4^Department of Biological, Geological and Environmental Sciences, University of Catania, Catania, Italy

**Keywords:** ALS, misfolded SOD1, mutant SOD1, N-terminal peptide, VDAC1

## Abstract

Mutations in superoxide dismutase (SOD1) are the second most common cause of familial amyotrophic lateral sclerosis (ALS), a fatal neurodegenerative disease caused by the death of motor neurons in the brain and spinal cord. SOD1 neurotoxicity has been attributed to aberrant accumulation of misfolded SOD1, which in its soluble form binds to intracellular organelles, such as mitochondria and ER, disrupting their functions. Here, we demonstrate that mutant SOD1 binds specifically to the N-terminal domain of the voltage-dependent anion channel (VDAC1), an outer mitochondrial membrane protein controlling cell energy, metabolic and survival pathways. Mutant SOD1^G93A^ and SOD1^G85R^, but not wild type SOD1, directly interact with VDAC1 and reduce its channel conductance. No such interaction with N-terminal-truncated VDAC1 occurs. Moreover, a VDAC1-derived N-terminal peptide inhibited mutant SOD1-induced toxicity. Incubation of motor neuron-like NSC-34 cells expressing mutant SOD1 or mouse embryonic stem cell-derived motor neurons with different VDAC1 N-terminal peptides resulted in enhanced cell survival. Taken together, our results establish a direct link between mutant SOD1 toxicity and the VDAC1 N-terminal domain and suggest that VDAC1 N-terminal peptides targeting mutant SOD1 provide potential new therapeutic strategies for ALS.

## Introduction

Amyotrophic lateral sclerosis (ALS) is a progressive and fatal neurodegenerative disease caused by the death of upper and lower motor neurons in the brain and spinal cord (Cleveland and Rothstein, 2001). The age of onset is typically between 50 and 60 years, followed by progressive paralysis and death 2–5 years after diagnosis (Mulder et al., 1986; Dorst et al., 2019). Most cases of ALS are sporadic and lack any apparent genetic linkage, although in 10% of cases, the disease is inherited in a dominant manner. About a fifth of these familial cases have been attributed to mutations in the gene encoding cytoplasmic Cu/Zn superoxide dismutase (SOD1) (Rosen et al., 1993).

To date, more than 180 different human SOD1 mutations have been identified throughout the length of the SOD1 protein that are directly linked to familial ALS (fALS),^1^ including active dismutase mutants, such as SOD1^G93A^ and SOD1^G37R^, and inactive dismutase mutants in which the mutation affects the metal-binding region, such as SOD1^G85R^ and SOD1^H46R^ (Abu-Hamad et al., 2017). The latter group of mutants are more unstable than are the former. Moreover, wild type human SOD1 (SOD1^WT^) can become misfolded and toxic, thus sharing an aberrant conformation with SOD1 mutants, when oxidatively modified (Tiwari et al., 2009; Bosco et al., 2010; Guareschi et al., 2012; Lim and Song, 2016; Medinas et al., 2018; Xu et al., 2018). The exact mechanism which drives motor neuron degeneration and disease progression remains unknown, although multiple hypotheses have been proposed to explain mutant SOD1-dependent toxic effects (Ilieva et al., 2009). These include ER stress, oxidative stress, glutamate-mediated excitotoxicity and mutant SOD1 misfolding and aggregation-induced pathology. Indeed, aggregation of misfolded SOD1 proteins is a common pathological observation among subjects with different SOD1 mutations and is, therefore, believed to be central to ALS pathogenesis (Bruijn et al., 1998; Wang et al., 2005; Prudencio et al., 2009; Abu-Hamad et al., 2017; Shvil et al., 2018).

Mitochondrial dysfunction has also been proposed as a major factor contributing to ALS pathology. SOD1 mutants affect various aspects of mitochondrial normal function, including fission and fusion, energy metabolism and transport (Ferri et al., 2006; Shi et al., 2010; Carri and Cozzolino, 2011; Magrane et al., 2012). Aberrant mitochondrial structures have been reported in both familial and sporadic ALS patients (Hirano et al., 1984a, b; Sasaki and Iwata, 1996, 2007), as well as in mutant SOD1 mouse models (Dal Canto and Gurney, 1994; Wong et al., 1995; Kong and Xu, 1998; Higgins et al., 2003). In addition, mitochondrial dysfunction and Ca^2+^ dysregulation has been reported in spinal cord and skeletal muscles of familial and sporadic ALS patients (Vielhaber et al., 1999; Echaniz-Laguna et al., 2002; Wiedemann et al., 2002; Dupuis et al., 2003), as well as in different ALS mouse models (Mattiazzi et al., 2002; Damiano et al., 2006; Nguyen et al., 2009).

Although predominantly a cytosolic protein, SOD1 is also found localized in other cellular compartments, including mitochondria. Both in mouse and rat models of ALS and post-mortem tissue samples from ALS patients, mutant SOD1 was found in fractions enriched for mitochondria derived only from affected but not unaffected tissues (Mattiazzi et al., 2002; Liu et al., 2004; Vijayvergiya et al., 2005; Bergemalm et al., 2006; Deng et al., 2006; Vande Velde et al., 2008). Moreover, a clear temporal correlation between disease progression and mitochondrial association was shown for different SOD1 mutants in rodent models (Liu et al., 2004). In addition, we have recently reported a clear inverse correlation between mutant SOD1 mitochondrial association in motor neuron-like NSC-34 cells and disease duration in patients carrying mutations in SOD1 (Abu-Hamad et al., 2017). Highly purified floated mitochondria coupled with protease accessibility has demonstrated deposition of mutant SOD1 on the cytoplasmic-facing surface of spinal cord mitochondria (Liu et al., 2004; Vande Velde et al., 2008). Sensitivity to proteolysis and immunoprecipitation with specific antibodies for misfolded SOD1 further demonstrated that misfolded species of SOD1 are associated with the outer mitochondrial membrane of the spinal cord (Vande Velde et al., 2008). In addition, mutant SOD1 was proposed to interact with other components of the outer mitochondrial membrane, including Bcl-2 (Pedrini et al., 2010) and the protein import machinery (Li et al., 2010), thus affecting the corresponding functions. Importantly, this was seen only for spinal cord mitochondria but not for mitochondria isolated from unaffected tissues (Israelson et al., 2010; Li et al., 2010). More specifically, direct binding of misfolded SOD1 to the voltage-dependent anion channel-1 (VDAC1) was previously shown, causing reduction of VDAC1 conductance and channel instability, leading to inhibition of VDAC1 transport of adenine nucleotides across the outer mitochondrial membrane (Israelson et al., 2010; Magri et al., 2016).

VDAC1, also known as the mitochondrial porin, is located at the outer mitochondrial membrane, where it assumes a crucial position controlling the metabolic cross-talk between the mitochondria and the rest of the cell, thus regulating the metabolic and energetic functions of mitochondria. VDAC1 is also a central player in mitochondria-mediated apoptosis and has been implicated in apoptotic-related functions, given its role as the target for pro- and anti-apoptotic Bcl2-family of proteins (Shimizu et al., 1999; Arbel and Shoshan-Barmatz, 2010) and due to its function in the release of apoptotic proteins from the mitochondrial inter membrane space (Tajeddine et al., 2008; Abu-Hamad et al., 2009). VDAC1, the main VDAC isoform, is composed of 19 transmembrane β-strands forming a membrane-embedded β-barrel and a flexible amphipathic 26-residue-long N-terminal domain lying inside the pore but able to translocate from within the pore to the channel surface (Geula et al., 2012). This mobility is important for controlling channel gating but also for interactions with pro- and anti-apoptotic proteins (Abu-Hamad et al., 2009; Arbel and Shoshan-Barmatz, 2010; Shoshan-Barmatz et al., 2010, 2015; Arbel et al., 2012; Geula et al., 2012). Importantly, cells expressing an N-terminally truncated form of VDAC1 are resistant to apoptosis (Abu-Hamad et al., 2009). These findings suggest that the VDAC1 N-terminal domain is required for interaction with VDAC1-associated proteins and apoptosis.

Here, we demonstrate the direct interaction of VDAC1 with mutant SOD1 and show that this interaction requires the VDAC1 N-terminal domain. Moreover, SOD1-mediated toxicity was prevented by synthetic VDAC1-N-terminal peptides. Finally, we show that a VDAC1 N-terminal peptide enhanced the survival of mutant SOD1^G93A^ motor neuron-like NSC-34 cells and mutant SOD1^G93A^ mouse embryonic stem cell-derived motor neurons. These findings point to VDAC1 N-terminal peptides as offering possible novel therapeutic strategies for ALS.

## Materials and Methods

### Materials

Bovine serum albumin (BSA), dithiothreitol (DTT), HEPES, leupeptin, phenylmethylsulfonyl fluoride (PMSF), propidium iodide (PI), sucrose and Tris were purchased from Sigma (St. Louis, MO, United States). Dulbecco’s modified Eagle’s medium (DMEM) was purchased from Gibco (Grand Island, NY, United States). The CellTiter 96 AQueous one-solution cell proliferation assay was purchased from Promega (Madison, WI).

### Peptides

The peptides used in this study (listed in Table 1) were synthesized by GL Biochem (Shanghai, China). The peptides were first dissolved in DMSO as a 40 mM solution and then diluted 20-fold in the appropriate buffer. Peptide concentrations were determined as described previously (Shteinfer-Kuzmine et al., 2018). The final concentration of DMSO in control and peptide-containing samples was ≤0.5%.

**TABLE 1:** Peptides used in this study.

**Peptide**	**Sequence**	**No. of AA**	**Molecular Mass, kDa**	**Calculated molar extinction coefficient, M^–1^**	**Purity, %**
1-26 N-Terminal	1-MAVPPTYADLGKSA- RDVFTKGYGFGL-26	26	2762	2980	>95
1-26 N-Ter-Antp	1-MAVPPTYADLGKSARDVFTKG YGFGL-26- RQIKIWFQNRRMKWKK	42	4991	13,980	97.72
1-20 N-Ter-Antp	1-MAVPPTYADLGKSARDVFTK-20-RQIKIWFQNRRMKWKK	36	4396	12,490	87.92
5-20 N-Ter-Antp	5-PTYADLGKSARDVFTK-20- RQIKIWFQNRRMKWKK	32	3997	12,490	87.39
10-20 N-Ter-Antp	10-LGKSARDVFTK-20- RQIKIWFQNRRMKWKK	27	3450	11,000	85.18
D-Δ(1-14) N-Ter-Antp	14-RDVFTKGYGFGL-26- RQIKIWFQNRRMKWKK	28	3558	12,490	95.79
LP3	159-ETAKSRVTQSNFAVGYKT-177	18	1987	1490	>85

*The VDAC1-based peptides used in this study with the amino acid sequence number, molecular mass, calculated molar extinction coefficient and purity are presented. The cell-penetrating sequence is underlined. The molar extinction coefficient was calculated based on amino acid composition using the following link: http://www.biomol.net/en/tools/proteinextinction.htm.*

### Protein Purification

Recombinant hSOD1^wt^, hSOD1^G93A^, and hSOD1^G85R^ were expressed in sf-9 cells and purified by hydrophobic interaction chromatography using phenyl-Sepharose 6 Fast Flow high sub (Amersham Biosciences), followed by ion exchange chromatography using a HiTrap Q-Sepharose anion exchange column (Amersham Biosciences), as described previously (Hayward et al., 2002).

### VDAC1 Purification

VDAC1 was purified from rat liver mitochondria using celite:hydroxyapatite and CMC chromatography, as previously described (Gincel et al., 2001). DNA sequences encoding full-length murine VDAC1 and N-terminally truncated VDAC1 (ΔN-VDAC1) lacking residues 1–26 were cloned into the pET21a vector (Novagen) using the *Nhe*I/*Xho*I sites. *Escherichia coli* BL21(DE3) cells were transformed with plasmid pET21a harboring the VDAC1 or ΔN-VDAC1 genes. Protein expression was induced for 3 h using 1 mM isopropyl-β-D-thiogalactopyranoside (IPTG; Sigma). Proteins were purified on agarose-packed nickel-nitrilotriacetic acid resin (Ni-NTA; Qiagen) in the presence of 8 M urea. Refolding of the eluted protein was performed essentially as described previously (Hiller et al., 2008). The refolded protein was further purified as above for mitochondrial VDAC1.

### Microscale Thermophoresis (MST)

Microscale thermophoresis analysis was performed using a NanoTemper Monolith NT.115 apparatus, as recently described (Wienken et al., 2010; Zillner et al., 2012). Briefly, purified wild type or mutant SOD1 proteins or mitochondria-purified VDAC1 were fluorescently labeled using the NanoTemper BLUE protein-labeling kit (NanoTemper Technologies, Munich, Germany). SOD1 was incubated for 20 min at 20°C in the dark with different concentrations of VDAC1-derived peptides (0.4–100 μM) in 10 mM HEPES buffer (pH 7.4) and then thermophoresis analysis was performed (light-emitting diode 20%, IR laser 20%). The results are presented as the bound fraction, calculated as follows: fraction bound 100 × (*F* − *F*min)/(*F*max − *F*min).

### VDAC1 Channel Reconstitution, Recording and Analysis

The reconstitution of recombinant WT or ΔN-VDAC1 into a planar lipid bilayer (PLB) prepared from soybean asolectin, and subsequent single channel current recordings and data analysis were carried out as described previously (Gincel et al., 2001). Currents were recorded under voltage-clamp conditions before and 5 min after the addition of 40 μg of recombinant hSOD1^wt^, hSOD1^G93A^, or hSOD1^G85R^ to the *cis* compartment using a Bilayer Clamp BC-535B amplifier (Warner Instrument, Hamden, CT, United States). Current amplitude histograms were prepared using AxoGraph X software. Relative conductance was determined as the average steady-state conductance at a given voltage normalized to the conductance at 10 mV, the maximal conductance. Relative conductance-voltage plots were prepared using Microsoft Excel software.

### Cell Treatment With VDAC1-Based Peptides, Cell Death and XTT Analyses

NSC-34 cells, a mouse motor neuron-like hybrid cell line, the A549 human lung adenocarcinoma and U-87MG human glioblastoma cell lines were incubated with the (1-26)N-Ter-Antp, D-(15-26)N-Ter-Antp, (1-20)N-Ter-Antp, (5-20)N-Ter-Antp, or (10-20)N-Ter-Antp peptides in the appropriate serum-free growth medium for 5 h at 37°C. Cells were harvested, and analyzed for cell death using propidium iodide (PI) staining and flow cytometry. For XTT, the CellTiter 96 AQueous one-solution cell proliferation assay was used to follow cell viability as described previously (Leyton-Jaimes et al., 2016).

Apoptotic cell death was also analyzed using Acridine Orange (AcOr)/ethidium bromide (EtBr) staining (McGahon et al., 1995). Cells in 24-well plates were washed with 200 μl PBS and 10 μl of a solution containing 100 mg/ml AcOr and 100 mg/ml EtBr in PBS was added. The cells were then visualized by fluorescence microscopy (ZOE fluorescence cell imager, Bio-Rad), images were recorded and cells at early and late apoptotic stages were counted.

### Immunostaining

For immunostaining, SH-SY5Y cells (4.5 × 10^4^) were grown on sterilized coverslips in 24-well plates. 30 h post-transfection, cells were fixed using 4% paraformaldehyde (PFA; diluted in PBS) for 15 min, and then washed 3 times with PBS (5 min each wash). Cells were then permeabilized with 0.3% Triton X-100 in PBS for 5 min followed by washing with PBS. Cells were then blocked for 1 h with blocking buffer (1% BSA free fatty acids diluted in PBS). Anti-VDAC1 polyclonal antibody (ab15895) and mouse anti misfolded SOD1 (B8H10, Medimabs) were incubated at room temperature for 1–2 h in a buffer of 1% BSA free fatty acids and 0.3% Triton-X100 in PBS. Following incubation with primary antibodies, cells were washed with PBS and incubated with fluorescent conjugated secondary Alexa Flour 488 anti-rabbit and Alexa flour 647 anti-mouse antibodies. The coverslips were carefully dried and mounted on slides using Immumount (Immumount^TM^, Thermo). After overnight drying, images were acquired on an Olympus IX81 confocal microscope.

### Mouse Embryonic Stem Cell (mESC) Cultures

Mouse embryonic stem cell lines harboring human mutant SOD1 (SOD1^G93A^) were a kind gift from Dr. Kevin Eggan (Harvard Stem Cell Institute). This cell line carries green fluorescent protein (GFP) under the control of the promoter for the motor neuron (MN)-specific transcription factor HB9 (*HB9*:GFP cells) (Di Giorgio et al., 2007). We used the SOD1^G93A^ mESC line to derive GFP^+^ MNs.

Motor neuron differentiation was induced as described previously with some modifications (Wichterle et al., 2002). Cells were cultured in ADFNB medium composed of advanced D-MEM/F12:Neurobasal (1:1), 0.1 mM 2-mercaptoethanol, and GlutaMAX supplement, B27 supplement, and N2 supplement all from Invitrogen, to form embryoid bodies (EBs), and supplemented with 0.1 μM of retinoic acid (RA, Sigma), as well as 0.5 μM of sonic hedgehog (Shh) agonist Ag1.3 (Curis) every second day.

For an *in vitro* differentiation assay, EBs were enzymatically dissociated after 7 days in culture with TrypLE Express (Gibco) and seeded on 0.01% poly-l-ornithine (Sigma) pre-coated coverslips followed by laminin (10 μg/ml, Sigma). Cells (5 × 10^4^) were seeded on coverslip in 24-well plates with ADFNB cell medium supplemented with of Ciliary neurotrophic factor (10 ng/ml, CNTF) and Glial cell-derived neurotrophic factor (GDNF; Miltenyi Biotec). During plating, 1-20N-Ter-Antp VDAC1-peptide was added to the cultures. Half of the medium was replaced with fresh medium every second day and at the indicated time points. The cultures were fixed in 4% paraformaldehyde in PBS.

### Determination of Neurite Outgrowth, Intersections Between Neurites and Survival

Stereological estimation of neurite lengths to evaluate neurite outgrowth in cultured cells was carried out as described previously (Ronn et al., 2000). The total neuritic length per cell was estimated by counting the number of GFP^+^ soma and neurite intersections with test lines of an unbiased counting frame superimposed on images of cell cultures obtained using a 20× objective (NA 0.75) of a Nikon Eclipse E800 epifluorescence microscope equipped with a Nikon DXM1200F CCD camera. The absolute length, *L*, of neurites per cell was subsequently estimated from the number of neurite intersections, *I*, per cell by means of the equation *L* = (*πd*/2)*I* describing the relationship between the number of neurite intersections and the vertical distance, *d*, between the test lines used.

### Statistical Analyses

Statistical comparisons between groups were performed by a two-tailed unpaired Student’s *t*-test. The mean ± SEM of results obtained from at least three independent experiments are presented. The significance of differences was calculated by a two-tailed Student’s *t*-test and is reported as ^∗∗^*p* < 0.01. Statistical comparisons between conditions in the mESC MN assays was performed by one-way ANOVA followed by Dunnett’s Multiple Comparison test against the control condition (Figure 6B) or Tukey’s Multiple Comparison test (Figures 6C,E). The confidence interval was stated at the 95% confidence level, placing statistical significance at *p* < 0.05. GraphPad Prism 6 was used for plotting data and statistical analysis.

## Results

### VDAC1 N-Terminal Domain and a VDAC1-Derived Peptide Specifically Interact With Mutant but Not Wild Type SOD1

**FIGURE 1:**
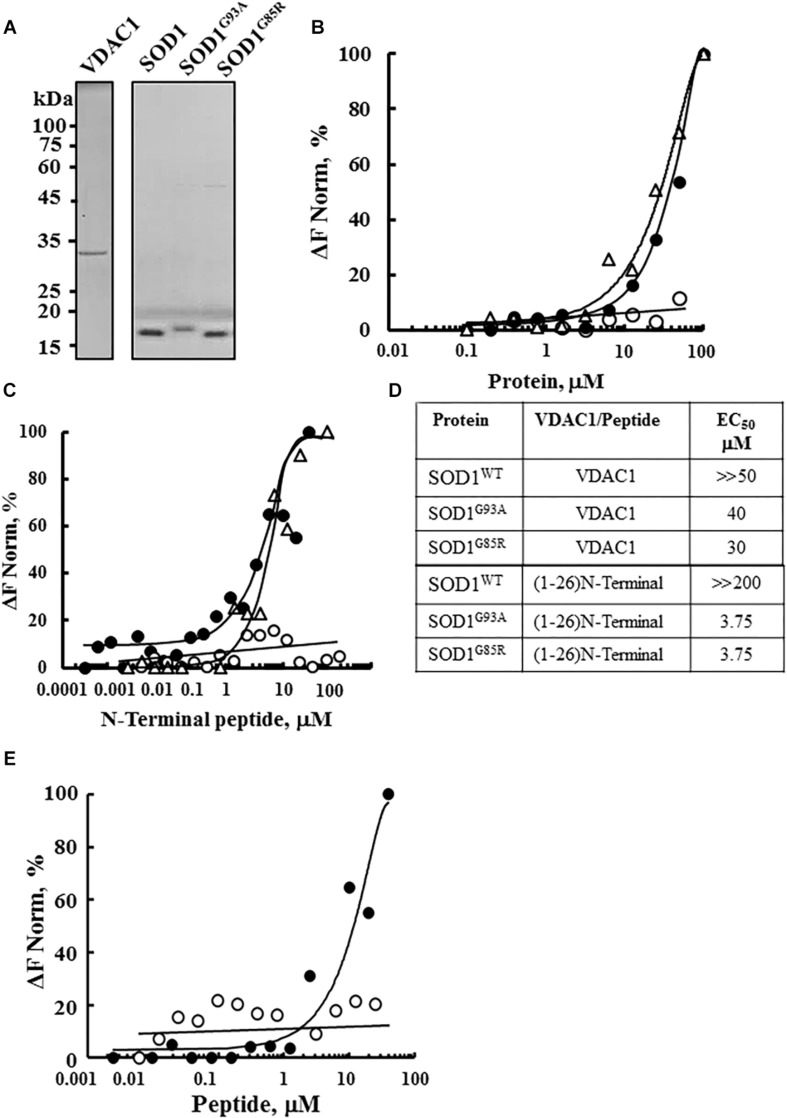
Interaction of wild type and mutant SOD1 with VDAC1 and VDAC1-N-terminal derived peptides. **(A)** Coomassie blue staining of mitochondrial VDAC1, and recombinant SOD1^WT^, SOD1^G93A^, and SOD1^G85R^ expressed in sf-9 cells, purified as described in the Methods section. Molecular weight standards are presented. **(B)** Purified VDAC1 was fluorescently labeled using a NanoTemper blue protein-labeling kit and incubated with SOD1^WT^ (∘), SOD1^G93A^ (∙), or SOD1^G85R^ (Δ; 0.1–100 μM). After 20 min of incubation, 3–5 μl aliquots were loaded into MST-grade glass capillaries (NanoTemper Technologies) and thermophoresis was measured with a NanoTemper Monolith-NT115 system. The percentage change in normalized fluorescence (ΔF Norm %) is plotted as a function of protein concentration. **(C)** Fluorescently labeled SOD1^WT^ (∘, 165 nM), SOD1^G85R^ (Δ, 230 nM) or SOD1^G93A^ (∙, 56 nM), was incubated with different concentrations of the VDAC1 N-terminal peptide (0.001–50 μM) in 10 mM HEPES buffer, pH 7.4 and analyzed for binding as in **(B)**. **(D)** Summary of the binding affinity (dissociation constants) of the peptide to SOD1^WT^, SOD1^G93A^ and SOD1^G85R^, as derived from a fitted curve of the percentage change in normalized fluorescence (ΔF Norm %) as a function of peptide and purified VDAC1 concentration. **(E)** Fluorescently labeled SOD1^G93A^ (675 nM) was incubated with the indicated concentrations of N-terminal (∙, 0.01–40 μM) or LP3 (∘, 6–40 μM) peptides in HEPES buffer, and binding was assayed as in **(B)**. *K*_*D*_ values were calculated using the mass action equation in the NanoTemper software from duplicate reads of triplicate experiments.

The interaction of purified WT and the SOD1 mutants SOD1^G93A^ and SOD^G85R^ with purified VDAC1 (Figure 1A) was assayed by MST (Figures 1B–E). MST measures any variation in the thermal movement of a fluorescently labeled binding partner. The subsequent fluorescence depletion in a heated spot of the protein solution is measured as a function of increasing interacting partner concentration, with dissociation constants (*K*_*D*_) values being derived from the depletion curves (Wienken et al., 2010; Figure 1D). Fluorescently labeled VDAC1 incubated with increasing concentrations of WT or mutant SOD1 (0–100 μM) showed that mutant SOD1^G93A^ and SOD1^G85R^ but not SOD1^WT^ bound to VDAC1 (Figure 1B).

Next, to identify the binding site for mutant SOD1 in VDAC1, we took advantage of different VDAC1-based peptides which we have developed and previously tested (Arzoine et al., 2008; Arbel and Shoshan-Barmatz, 2010). As the N-terminal domain of VDAC1 has been the shown to interact with several proteins, such as hexokinase, Bcl-2 and Bcl-xL (Arzoine et al., 2008; Arbel and Shoshan-Barmatz, 2010; Arbel et al., 2012), we tested whether a synthetic N-terminal peptide interacts with SOD1. Accordingly, fluorescently labeled mutant SOD1^G93A^ or SOD1^G85R^ protein was incubated with increasing concentrations of the synthetic VDAC1 N-terminal peptide and changes in fluorescence were monitored (Figure 1C). By plotting the percentage change of normalized fluorescence (ΔF Norm %) as a function of peptide concentration, a fitted curve yielded dissociation constants (*K*_*D*_) for the three versions of SOD1 (Figure 1D). The results showed that the VDAC1 N-terminal peptide bound both mutant SOD1^G93A^ and SOD1^G85R^, but not to SOD1^WT^ (Figures 1C,D), indicating that these mutants interact specifically with the N-terminal region.

The specificity of the N-terminal peptide to mutant SOD1 was demonstrated by testing the binding of another VDAC1-derived peptide, LP3. This peptide, representing the sequence of a VDAC1 loop facing the cytosol (Arzoine et al., 2008), showed significantly lower binding to mutant SOD1 than did the (1-26)-N-terminal peptide (Figure 1E). These results show that VDAC1 and the N-terminal VDAC1 peptide specifically interact with mutant SOD1^G93A^ and SOD1^G85R^.

### Mutant but Not Wild Type SOD1 Interacts With VDAC1 and Reduces Its Channel Activity

**FIGURE 2:**
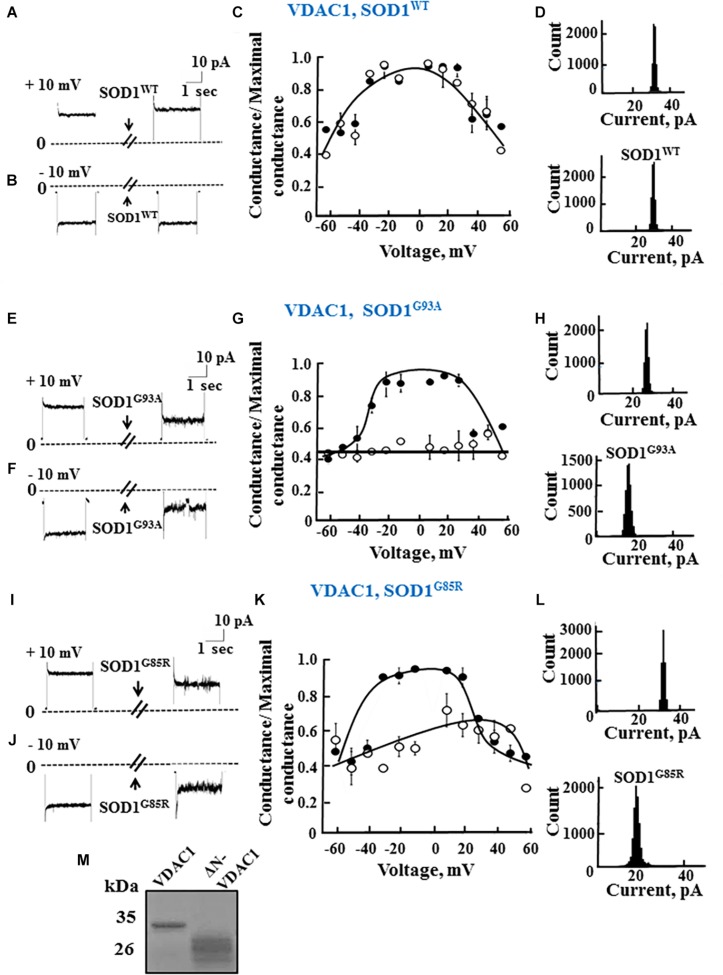
Mutant SOD1 but not wild type SOD1 interacts with VDAC1 and inhibits channel conductance. **(A,B)** Recombinant full length VDAC1 purified from *E. coli* was reconstituted into a PLB and channel currents through VDAC1, in response to a voltage step from 0 to 10 mV **(A)** or to –10 mV **(B)**, before and 15–20 min after addition of 40 μg/ml (final concentration) of SOD1 were recorded. **(C)** VDAC1 relative conductance as a function of voltage in a 60 to –60 mV step before (∙) and after addition of SOD1^WT^ (∘). Relative conductance (conductance/maximal conductance) was determined as the average steady-state conductance at a given voltage normalized to the conductance at 10 mV, considered the maximal conductance. **(D)** Histogram of VDAC1 current amplitudes at 10 mV before and after addition of SOD1^WT^. **(E–H)** Similar experiments as in **(A–D)**, except that mutant SOD1^G93A^ was used. **(I–L)** Similar experiments as in **(A–D)**, except that mutant SOD1^G85R^ was used. **(M)** Coomassie blue staining of recombinant full length and residue 1-26-truncated VDAC1 (ΔN-VDAC1).

To test whether mutant hSOD1^G93A^ and hSOD1^G85R^ binding to VDAC1 affects VDAC1 function, as well as the requirement of the N-terminal domain for such binding, full length and N-terminally truncated VDAC1 were expressed in *E. coli*, purified (Figure 2M) and reconstituted into a PLB as described previously (Gincel et al., 2001).

Single-channel conductance under voltage-clamp conditions was measured as a function of time, reflecting ions passing through the channel in response to an applied voltage gradient. Current-time traces recorded at −10 or 10 mV from purified recombinant full length VDAC1 showed a stable full open state that was maintained for extended periods. Addition of hSOD1^WT^, even at the highest concentration (60 μg/ml), had no effect on the current (Figures 2A,B). VDAC1 showed a bell-shaped relative conductance curve as function of the voltage, with hSOD1^WT^ having no effect on VDAC1 channel conductance at all tested voltages, i.e., −60 to +60 mV (Figure 2C). This was also revealed in the current amplitude histograms (Figure 2D), which showed a single channel conductance of 32 pA, at 10 mV.

In contrast to hSOD1^WT^, both mutant hSOD1^G93A^ and hSOD1^G85R^ reduced the channel conductance of bilayer-reconstituted VDAC1 at all tested voltages and decreased the current amplitude histograms (Figures 2E–L). hSOD1^G93A^ was found to be more effective in reducing VDAC1 conductance than was hSOD1^G85R^ when added at the same concentration, resulting in 57 and 40% inhibition of channel conductance, respectively (Table 2).

**TABLE 2:** Summary of the effects of SOD1^WT^ and mutant SOD1 on the conductance of VDAC1 and ΔN-VDAC1 reconstituted in planar lipid bilayer.

**Protein**	**Conductance, nS**	
	
	**VDAC1**	**VDAC1 + SOD1**
SOD1^WT^	3.79 ± 0.11	3.84 ± 0.1
SOD1^G93A^	3.51 ± 0.005	1.65 ± 0.33
SOD1^G85R^	3.65 ± 0.14	2.3 ± 0.35

	**ΔN-VDAC1**	**ΔN-VDAC1 + SOD1**

SOD1^WT^	3.88 ± 0.11	3.69 ± 0.13
SOD1^G93A^	3.39 ± 0.19	3.45 ± 0.01
SOD1^G85R^	3.65 ± 0.14	3.92 ± 0.08

**FIGURE 3:**
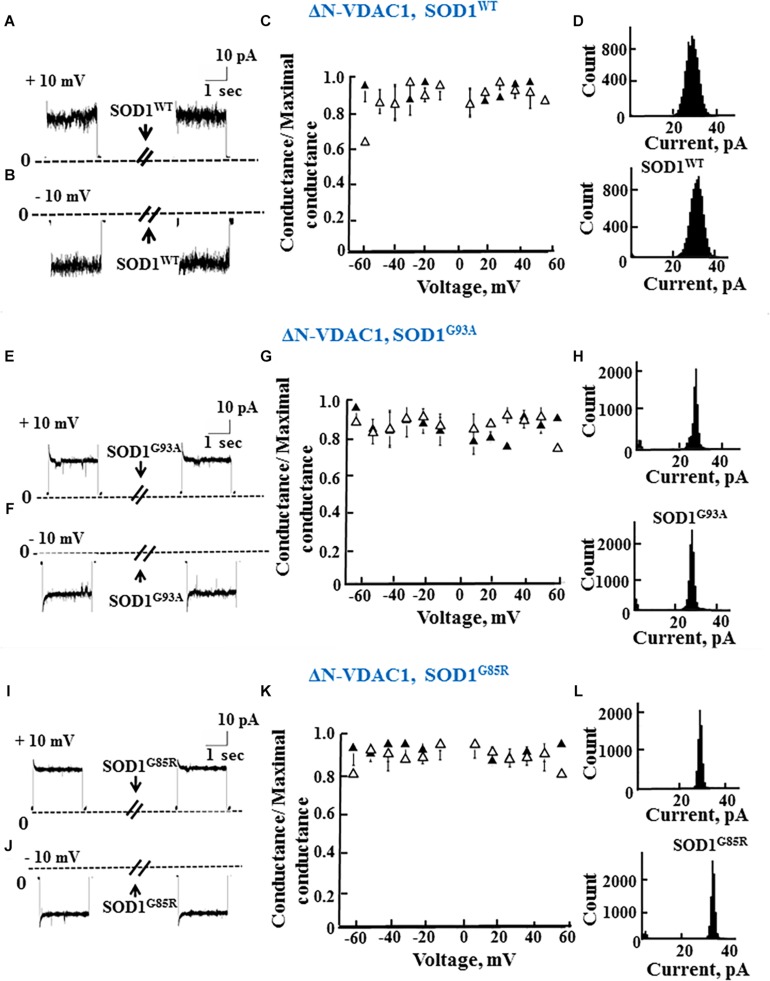
The N-terminal domain of VDAC1 is required for mutant SOD1 interaction with VDAC1 and inhibition of channel conductance. **(A,B)** Currents passing through bilayer-reconstituted recombinant N-terminally truncated VDAC1 (ΔN-VDAC1) were recorded in response to voltage step from 0 to 10 mV **(A)** or –10 mV **(B)** before and 15–20 min after the addition of 40 μg/ml (final concentration) of SOD1^WT^. **(C)** Relative conductance of ΔN-VDAC1 as a function of voltage in a step from 60 to –60 mV before (▲) and after addition of SOD1^WT^ (Δ). Relative conductance (conductance/maximal conductance) was determined as the average steady-state conductance at a given voltage normalized to the conductance at 10 mV, taken as the maximal conductance. **(D)** Histogram of VDAC1 current amplitudes at 10 mV before and after addition of SOD1^WT^. **(E–H)** Similar experiments as in **(A–D)**, except that mutant SOD1^G93A^ was used. **(I–L)** Similar experiments as in **(A–D)**, except that mutant SOD1^G85R^ was used.

*Results are the conductance (1 M NaCl, pH 7.4, at 10 mV), calculated from experiments as in Figures 2, 3 presented as mean ± SE (*n* = 3).*

As reported previously (Prezma et al., 2013; Shteinfer-Kuzmine et al., 2018), VDAC1 lacking the N-terminal 26 residues (ΔN-VDAC1) showed no voltage-dependent gating, with hSOD1^WT^ having no effect on channel conductance at all voltages tested or on the channel current amplitude histograms (Figures 3A–D). Moreover, and in contrast to what was observed upon SOD1 mutants interaction with VDAC1 (see Figures 2E–L), hSOD1^G93A^ or hSOD1^G85R^ had no effect on ΔN-VDAC1 conductance at all tested voltages (Figures 3E–L). These results thus suggest that VDAC1 N-terminal is important for binding of hSOD1^G93A^ and hSOD1^G85R^ to VDAC1.

The summary of the effects of SOD1^WT^, hSOD1^G93A^, and hSOD1^G85R^ on the channel conductance of VDAC1 and ΔN-VDAC1 is presented in Table 2.

### Cell-Penetrating VDAC1 N-Terminal Peptides Inhibit Cell Death of NSC-34 Cells as Induced by Mutant SOD1^G93A^

**FIGURE 4:**
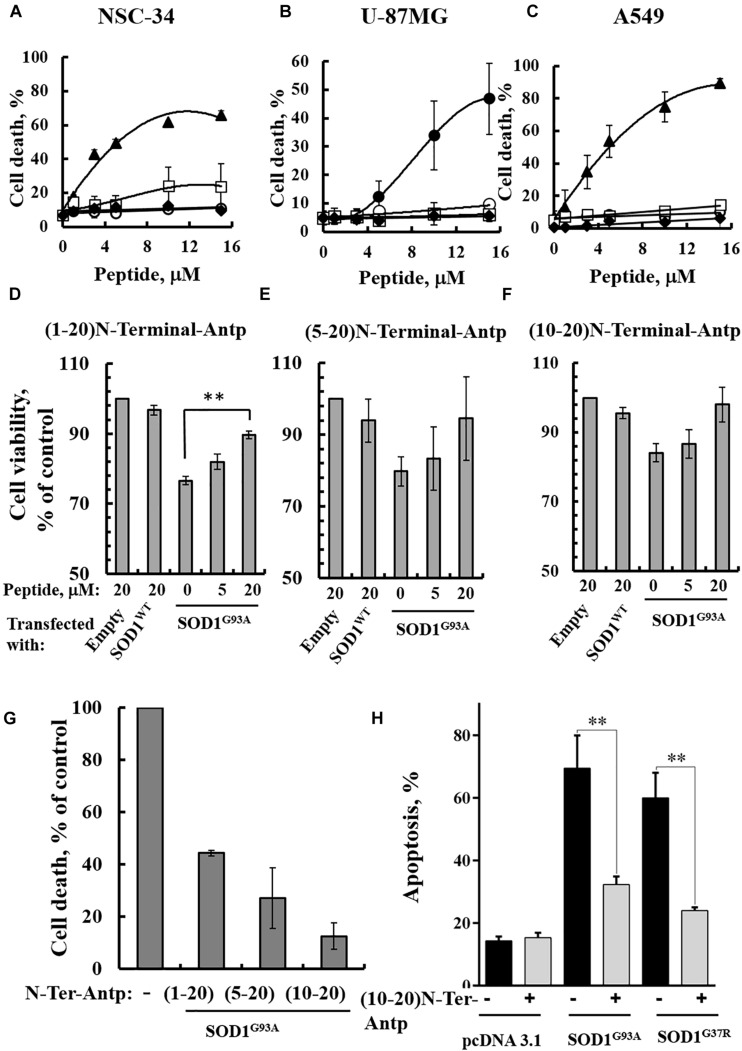
VDAC1 N-terminal peptides inhibit the cell death of NSC-34 cells mediated by mutant SOD1^G93A^. NSC-34 **(A)**, U-87MG **(B)**, or A549 **(C)** cells were incubated with the indicated concentrations of (1-26)N-Ter-Antp (∙), D-(15-26) (19-26)N-Ter-Antp (▲), (1-20)N-Ter-Antp (∘), (5-20)N-Ter-Antp (□) or (10-20)N-Ter-Antp (◆) peptide for 5 h. Cell death was analyzed using PI staining and flow cytometry. **(D–F)** NSC-34 cells were transfected to express human SOD1^WT^, the human mutant SOD1^G93A^, or neither (empty), in each case either without or with addition of increasing concentrations of the indicated VDAC1 N-terminal-derived peptide for 5 h. Cell viability analysis was performed with the CellTiter 96 AQueous one-solution cell proliferation assay with ELISA at 490 nm. **(G)** The rescuing effects of the VDAC1 N-terminal-derived peptides are shown as a percentage of cell death. The significance of quantitative analysis of triplicates of different biological repeats (*n* = 3) was performed by Student’s *t*-test; ^∗∗^*P* < 0.01. **(H)** SH-SY5Y cells (4.5 × 10^4^ cells/well in 24-well plates) were transfected with an empty plasmid or a plasmid encoding for mutant SOD1^G93A^ or SOD1^G37R^. Twenty-four hours post-transfection, the cells were incubated for 5 h with (10-20)N-Ter-Antp peptide (20 μM) and then analyzed for apoptosis using acridine orange and ethidium bromide staining, as described previously (McGahon et al., 1995). Fluorescence microscopy images were analyzed and about 100 to 300 cells were counted for each treatment in representative microscopic fields. The significance of quantitative analysis of triplicates of different biological repeats (*n* = 3) was performed by one-way Anova; ^∗∗^*P* < 0.01.

In our previous study (Shteinfer-Kuzmine et al., 2018), several novel VDAC1 N-terminal-derived peptides were designed and tested for their ability to induce cell death in cancer cells. Here, we sought to determine whether any of these peptides does not induce cell death yet can interact with misfolded mutated SOD1 and protect against SOD1^G93A^-mediated cell death in neuronal cultures. Accordingly, we assessed the effects of synthetic cell-penetrating VDAC1-N-terminal-derived peptides in inducing cell death in NSC-34 cells, a mouse motor neuron-like hybrid cell line, and in the U-87MG and A549 cancer cell lines (Figures 4A–C). We considered five cell-penetrating peptides derived from the VDAC1-N-terminal domain in these studies: (1) (1-26)-N-Ter-Antp peptide composed of the first 26 residues of the VDAC1 sequence fused to the cell-penetrating Antp (penetrating) sequence, a 16 residue long sequence from the *Drosophila* antennapedia-homeodomain; (2) D-(15-26)-N-Ter-Antp, a peptide like (1-26)-N-Ter-Antp but shortened by 13 amino acids from the N-terminus, and presenting all residues in the D configuration; (3) (1-20)-N-Ter-Antp peptide, again a peptide like (1-26)-N-Ter-Antp but shortened by six amino acids at the C-terminus; (4) (5-20)-N-Ter-Antp peptide, a peptide like (1-20)-N-Ter-Antp peptide but shortened by four amino acids at the N-terminus; and (5) (10-20)-N-Ter-Antp peptide, a peptide like (1-20)-N-Ter-Antp peptide but shortened by nine amino acids at the N-terminus of the peptide (Table 1).

The results showed that (1-26)-N-Ter-Antp and D-(15-26)-N-Ter-Antp peptides induced massive cell death in NSC-34 (Figure 4A), U-87MG (Figure 4B), and A549 (Figure 4C) cells, as analyzed using PI and flow cytometry analysis. However, shortening the (1–26)-N-Ter-Antp by deleting six residues from the C-terminus of the peptide, including the GXXXG motif, to generate the (1-20)-N-Ter-Antp, (5-20)-N-Ter-Antp and (10-20)-N-Ter-Antp peptides, yielded peptides that could not induce cell death (Figures 4A–C).

To test *in vitro* whether the different shortened versions of the VDAC1 N-terminal peptide could prevent mutant SOD1 toxicity in neurons, NSC-34 cells were transfected to express SOD1^WT^ or mutant SOD1^G93A^ protein and then treated with or without the above indicated VDAC1 N-terminal peptides. Cell viability was quantified using the XTT assay and apoptosis using acridine orange and ethidium bromide staining. Whereas SOD1^WT^ had not effect on cell viability, expressing SOD1^G93A^ reduced cell viability by 25–30%. The presence of VDAC1 N-terminal peptides reduced the toxic effect of SOD1^G93A^ in a concentration-dependent manner (Figures 4D–G). Importantly, this effect was observed using the three non-cell death-inducing VDAC1-derived N-terminal peptides, i.e., the (1-20)-N-Ter-Antp, (5-20)-N-Ter-Antp and (10-20)-N-Ter-Antp peptides.

In order to show that the rescue effect of the N-terminal peptide is not specific for mutant SOD1^G93A^, but a general effect, we have expressed two different dismutase active mutants SOD1^G93A^ or SOD1^G37R^ to induce cell death, increasing it from 17% in the control plasmid-transfected cells to 67 and 70%, in cells expressing mutant SOD1^G93A^ or SOD1^G37R^, respectively. Incubation with the (10-20)-N-Ter-Antp peptide, decreased SOD1^G93A^ and SOD1^G37R^-mediated cell death by 68 and 81%, respectively (Figure 4H).

**FIGURE 5:**
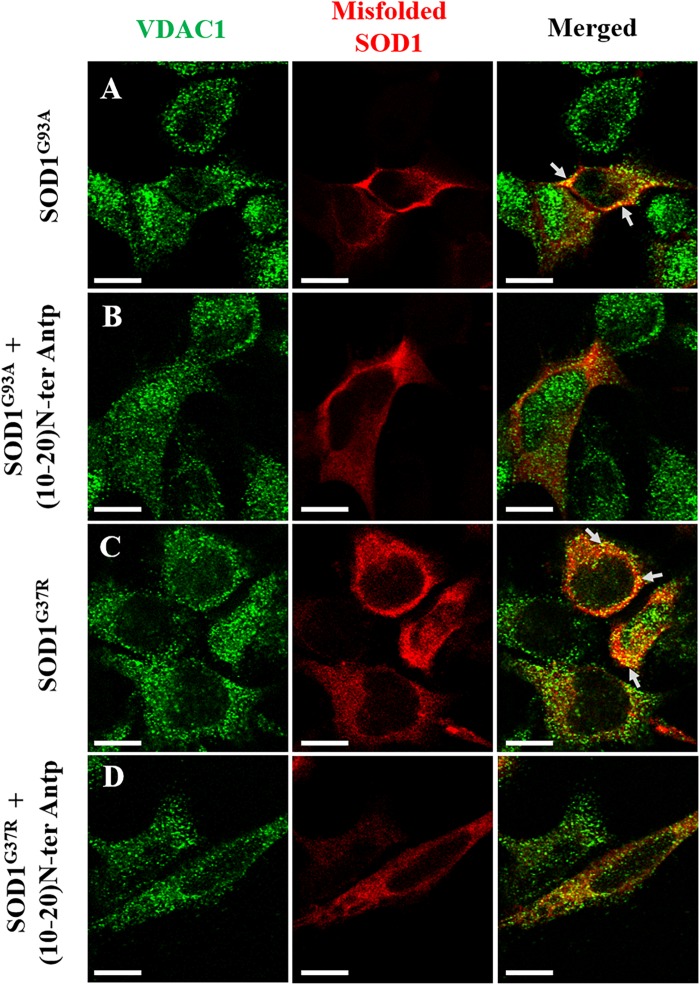
The VDAC1 N-terminal peptide induces detachment of misfolded SOD1 from its mitochondrial binding site. Co-localization of VDAC1 and misfolded SOD1 in SH-SY5Y cells was assayed by indirect immunofluorescence with antibodies for VDAC1 along with simultaneous identification of misfolded SOD1 (with the B8H10 antibody). Twenty-four hours post-transfection, the cells were incubated for 5 h with (10-20)N-Ter-Antp peptide (20 μM) and then analyzed for co-localization using a confocal microscope. Representative images of SH-SY5Y cells transfected to express human mutant SOD1^G93A^ **(A)** or SOD1^G37R^ **(C)** in each case either without **(A,C)** or with **(B,D)** addition of the (10-20)N-Ter-Antp peptide are shown. Scale bar, 10 μm.

In addition, to test whether the N-terminal VDAC1-based peptide could promote misfolded SOD1 detachment from the mitochondria and more specifically from VDAC1, SH-SY5Y neuronal cells were transfected to express two different mutated SOD1^G93A^ or SOD1^G37R^ proteins and then treated with or without the VDAC1 (10-20)-N-Ter-Antp peptide. Co-localization of misfolded SOD1 with VDAC1 was determined by immunostaining analysis using an anti-VDAC1 antibody that does not target the N-terminus region of the protein, and the B8H10 antibody specifically recognizing misfolded SOD1. In cells transfected with SOD1^G93A^ or SOD1^G37R^, VDAC1 staining is punctuated as expected for mitochondrial localization, while misfolded SOD1 shows both punctuated staining but mostly diffused, indicating that part of the misfolded SOD1 protein is mitochondria bound (Figures 5A,C). Yet, a clear co-localization of misfolded SOD1 with VDAC1 can be observed (Figures 5A,C). However, this co-localization was greatly eliminated in cells subjected to treatment with (10-20)-N-Ter-Antp peptide (Figures 5B,D).

### A VDAC1 N-Terminal Peptide Enhances the Survival of Mouse Embryonic Stem Cell-Derived Motor Neurons Expressing SOD1^G93A^

The effects of increasing concentrations of the VDAC1-based (1-20)-N-Ter-Antp peptide on three characteristics of SOD1^G93A^-expressing ESC-derived motor neurons were analyzed *in vitro*. Specifically, we assessed neurite outgrowth, MN survival shortly after induction of final differentiation and survival of maturing SOD1^G93A^-expressing MNs. To follow neuronal development and remodeling of neuronal extensions, we determined neurite outgrowth in cell cultures by tracing neurites and their branches using the stereological procedure, as described previously (Ronn et al., 2000). SOD1^G93A^-expressing MNs grown in relatively pure cultures die rapidly over the 1st week (Aggarwal et al., 2017). Therefore, the first two assays were performed over the first 2 days of culture. This period is characterized by small to minimal loss of MNs, with the exception of those cells that fail to attach or which die immediately. The third assay was performed after the onset of cell death, when around 50% of cells had died.

**FIGURE 6:**
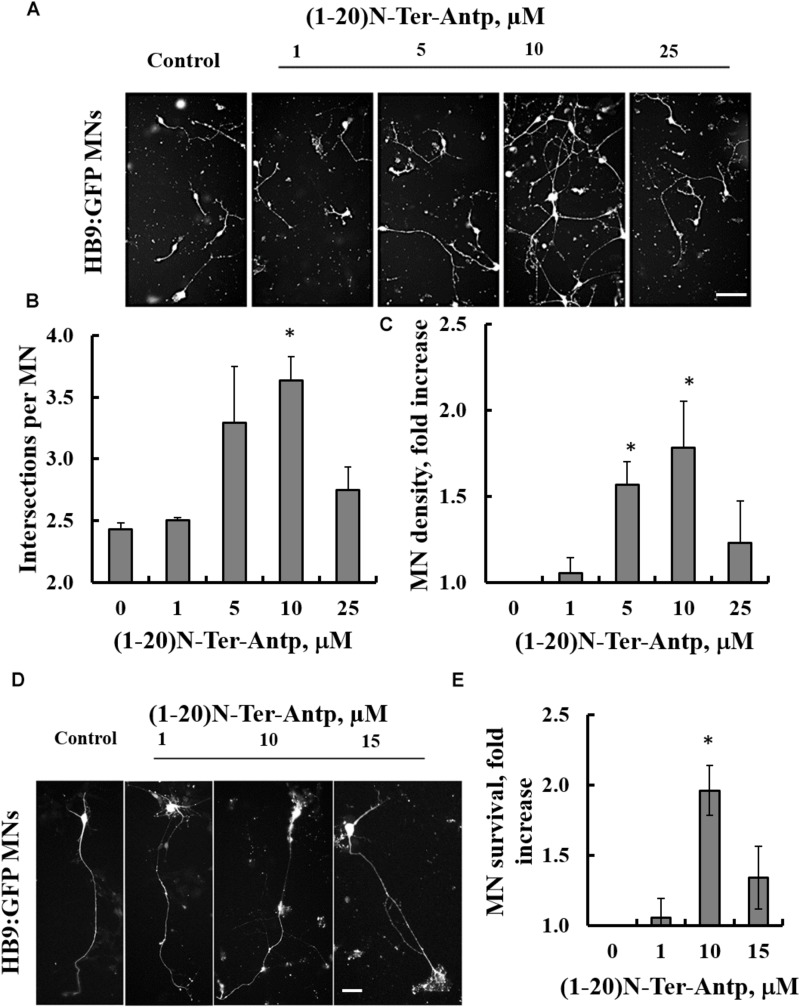
The VDAC1 N-terminal peptide ameliorates SOD1^G93A^-mediated motor neuron toxicity. SOD1^G93A^-expressing mESC-derived MNs were incubated with the indicated concentrations of (1-20)N-Ter-Antp peptide for 1–4 days following initiation of final differentiation. **(A,B)** (1-20)N-Ter-Antp at a concentration of 10 μM significantly increased MN neurite outgrowth over the first 24 h. Scale bar, 100 μm. **(C)** Twenty-four hours after the initiation of differentiation, 5 and 10 μM of (1-20)N-Ter-Antp peptide significantly increased the number of MNs present in the culture. **(D)** (1-20)N-Ter-Antp at a concentration of 10 μM significantly increased the number of surviving MNs over the course of final differentiation. After 96 h, MNs showed the typical cellular morphology of maturing neurons with extension of a singular long process and branching resembling a dendritic tree. GFP fluorescence indicates the expression of the motor neuron-specific transcription factor HB9. Gross cellular morphology of surviving MNs remained unaffected by the (1-20)N-Ter-Antp peptide. Scale bar, 25 μm. **(E)** Quantification of MNs survival. For all assays, results are expressed as the mean ± SEM (*n* = 3). ^*^P < 0.05.

Images showing the effects of the N-terminal peptide on neurite outgrowth of mESC-derived SOD1^G93A^-expressing MNs (Figures 6A,D), as well as quantification of neurite outgrowth (Figure 6B), are presented. Cells were incubated with the indicated concentrations of the peptide and analyzed 24 h later. The results showed that adding the peptide at a 10 μM concentration significantly (*p* < 0.05) improved neurite outgrowth of SOD1^G93A^-expressing MNs (Figures 6A,B). The effect of the peptide on the survival of mESC-derived SOD1^G93A^-expressing MNs was analyzed 24 h after plating. The results showed that when added at concentrations of 5 or 10 μM, the peptide significantly (*p* < 0.05) improved MN density (Figures 6A,C). The reduced effect of the peptide at higher concentrations (25 μM) may result from other non-specific interactions. Moreover, the VDAC1 N-terminal peptide extended survival of the mESC-derived SOD1^G93A^-expressing mature MNs (Figures 6D,E). When five cultures were incubated with the indicated peptide concentration for 96 h, it was seen that the peptide significantly (*p* < 0.05) improved MN survival when administered at a 10 μM concentration (Figures 6D,E). Thus, the peptide improved survival of SOD1^G93A^-expressing MNs by twofold.

## Discussion

The results of the present study contribute to the better understanding of mutant SOD1-mediated mitochondrial dysfunction and cellular toxicity with relevance to ALS pathogenesis. Our results demonstrate that SOD1 mutants bind directly and selectively to the N-terminal domain of VDAC1. Both dismutase-active SOD1^G93A^ and dismutase-inactive SOD^G85R^ mutants, but not wild type SOD1, bind to the VDAC1 N-terminal region. Moreover, we demonstrated that different versions of an N-terminal peptide suppressed mutant SOD1 toxicity in motor neuron-like NSC-34 cells expressing mutant SOD1 in a concentration-dependent manner and enhanced the survival of SOD1^G93A^-expressing mESC-derived motor neurons.

### Mutant SOD1 Interacts With VDAC1 to Mediate Mitochondrial Dysfunction

The direct interaction of mutant SOD1 with VDAC1 was previously determined by immunoprecipitation using anti-SOD1, anti-VDAC1 and anti-misfolded SOD1 antibodies together with mutant SOD1 from rat spinal cord tissues or using purified proteins in a lipid bilayer system (Israelson et al., 2010; Magri et al., 2016). Now, we extended these findings and showed using both MST and VDAC1 channel conductance that the dismutase-active SOD1^G93A^ mutant or the dismutase-inactive SOD1^G85R^ mutant but not SOD1^wt^ specifically interact with VDAC1.

Binding of misfolded SOD1 species to mitochondrial membranes was shown to disrupt transport of metabolites required for oxidative phosphorylation, reduce membrane potential, and the activity of electron transport chain complexes (Liu et al., 2004), and to the generation of reactive oxygen (ROS) or nitrogen species with damaging effects on respiratory chain complexes (Martin et al., 2007). All these effects can be produced by interactions of mutant SOD1 with VDAC1, which mediates the transport of metabolites, ions (including Ca^2+^) and ROS (Shoshan-Barmatz et al., 2010).

### VDAC1 Interacts With Mutant SOD1 Through the VDAC1 N-Terminal Domain

We identified the binding sites mediating VDAC1-mutant SOD1 interactions by showing that SOD1^G93A^ and SOD1^G85R^ do not bind the VDAC1 N-terminally truncated protein, but interact with the VDAC1-derived N-terminal peptide, suggesting that mutant SOD1 interacts with the VDAC1 N-terminal domain to mediate its cell toxic effects. This finding is not surprising as the N-terminal domain of VDAC1 was shown to be the interaction site for many proteins (Arzoine et al., 2008; Abu-Hamad et al., 2009; Arbel and Shoshan-Barmatz, 2010; Arbel et al., 2012; Geula et al., 2012) and to possess an ATP-binding site (Yehezkel et al., 2007). The first eight amino acids of the VDAC1-N-terminal domain are hydrophobic in nature, thus providing a natural possible site of contact with misfolded SOD1. Wild type recombinant SOD1 remains soluble, whereas mutations in the SOD1 protein leads to exposure of certain hydrophobic residues normally buried with the protein core. These structural changes lead to misfolding and aggregation of mutant SOD1 proteins via exposure of hydrophobic residues, such that they interact with intracellular membranes, such as mitochondria, ER and others (Israelson et al., 2015).

The interaction of misfolded SOD1 with VDAC1 is further suggested by the co-localization of two different SOD1 mutants with VDAC1 at the mitochondria (Figure 5). Furthermore, the N-terminal-derived peptide decreasing the extent of this co-localization, points to VDAC1-N-terminus as the misfolded SOD1 interaction site. Surprisingly, we have noticed a tendency for a nuclear localization of VDAC1 in cells accumulating misfolded SOD1. This phenomenon is not clear and should be further investigated.

The VDAC1 N-terminal region is proposed to move within the channel pore (Hiller and Wagner, 2009) and to translocate from the internal pore to the channel surface (Geula et al., 2012), allowing it to interact with cytosolic proteins. The multiple glycine residues (^21^GlyTyrGlyPheGly^25^) following this domain represent a GXXXG motif that connects the N-terminal domain to β-strand 1, and confer the flexibility required for N-terminal domain translocation out of the channel pore (Geula et al., 2012). The GXXXG motif has been shown to be involved in dimerization in proteins such as glycophorin A (Gerber and Shai, 2001), human carbonic anhydrase (Whittington et al., 2001), yeast ATP synthase (Saddar and Stuart, 2005), carnitine palmitoyltransferase (Jenei et al., 2009), and others. In VDAC1, this motif is not required for VDAC1 dimerization but it might be involved in interaction with VDAC1-associated proteins (Geula et al., 2012). Interestingly, SOD1 contains three GXXXG (residues 11–16, 32–36, and 36–41) and two GXXXXG motifs (residues 51–56 and 56–61), but their importance for the interaction of VDAC1 with mutant SOD1 proteins is unknown.

**FIGURE 7:**
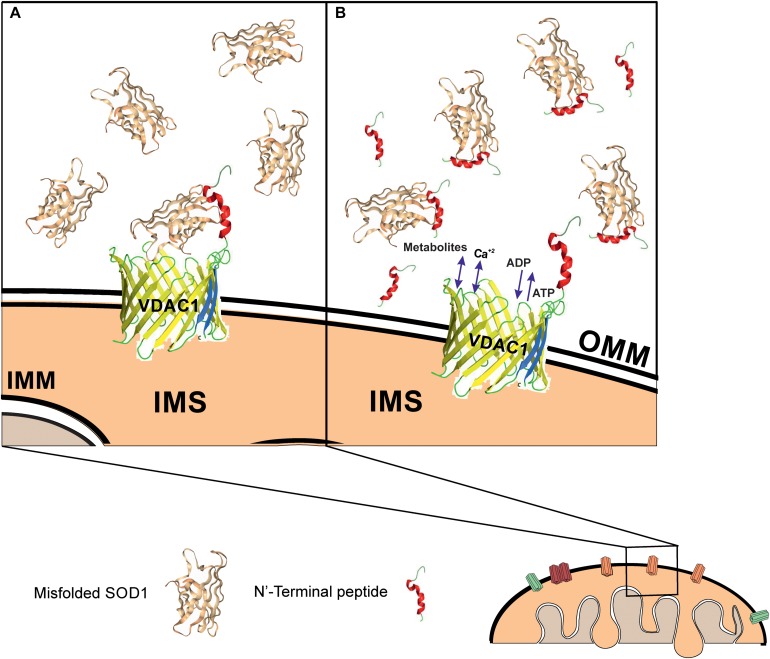
Mutant SOD1 binds to VDAC1 and inhibits VDAC1 activities, with addition of a VDAC1 N-terminal peptide preventing such inhibition. Schematic model showing mutant SOD1 binding to VDAC1, with the N-terminal peptide serving as a decoy. **(A)** Mutant SOD1 is proposed to bind to the VDAC1 N-terminal domain and inhibit VDAC1 conductance, thereby suppressing both influx and efflux of different mitochondrial metabolites and ions, including Ca^2+^, and ROS. This reduction in metabolite flux results in reduced energy production and increased oxidative stress, leading to mitochondrial dysfunction and cell death. **(B)** VDAC1 N-terminal-derived peptides bind mutant SOD1 and prevent its association with VDAC1, thereby preventing mitochondria dysfunction. The N-terminal peptide thus provides a new therapeutic approach for inhibiting mutant SOD1 toxicity in ALS. OMM and IMM indicate outer and inner mitochondrial membrane, respectively while IMS indicates, the intermembrane space.

In a PLB, reconstituted VDAC1 but not the N-terminally truncated protein bound a misfolded SOD1 mutant but not wild type SOD1, leading to reduced VDAC1 conductance. It is very likely that the exposure of residues in mutant SOD1 proteins that are normally hidden leads to increased interactions between SOD1 mutants and the VDAC1 N-terminal domain. Such association would reduce the overall transit of ions through the VDAC1 pore, thereby leading to the observed reduction in VDAC1 conductance. This interaction is expected to also reduce ATP, ADP, metabolite and ROS transport, in turn leading to an inhibition of cell growth and induction of mitochondrial dysfunction and cell toxicity (Figure 7).

### VDAC1 N-Terminal Peptides Inhibit Cell Death of NSC-34 Cells Expressing Mutant SOD1^G93A^ and Enhance Survival of Mouse SOD1^G93A^-Expressing mESC-Derived Motor Neurons

We have shown here that different versions of the N-terminal peptide are able to suppress the toxicity of motor neuron-like NSC-34 cells expressing mutant SOD1^G93A^ in a concentration-dependent manner. For these experiments, we used short versions of the VDAC1 N-terminal domain peptide which lack the GXXXG motif, as this domain is required for N-terminal peptide-induced cell death (Figures 4A–C) but not for the interaction with mutant SOD1 (Figures 4D–F).

In contrast to mutant SOD1 that by binding to VDAC1 N-terminus induces apoptosis, the binding of apoptosis regulatory proteins such as Bcl-2, Bcl-xL and hexokinase to VDAC1 protects against apoptosis (Arzoine et al., 2008; Abu-Hamad et al., 2009; Arbel and Shoshan-Barmatz, 2010; Arbel et al., 2012; Prezma et al., 2013; Shteinfer-Kuzmine et al., 2018). This protection is mediated via their binding to the N-terminus (Arzoine et al., 2008; Arbel and Shoshan-Barmatz, 2010; Arbel et al., 2012), with Δ(1-24)hVDAC1 showing decreased binding to Bax or Bcl-xL relative to VDAC1 or Δ(1-12) VDAC1 (Shi et al., 2003). Accordingly, interfering with their binding to VDAC1 resulted in apoptosis induction. In addition, the N-terminal peptide lacking 21–26 amino acids, used in the current study, lost its ability to induce apoptosis (Prezma et al., 2013; Shteinfer-Kuzmine et al., 2018). Thus, these results suggest that the peptides serve as decoy to compete with VDAC1 for misfolded SOD1 binding, thereby protecting against its cytotoxicity.

Finally, experiments with mESC-derived SOD1^G93A^-expressing MNs showed that the (1-20)-N-Ter-Antp VDAC1-based peptide significantly improved neurite outgrowth and the survival of SOD1^G93A^-expressing MNs in a concentration-dependent manner (with an optimal peptide concentration of 10 μM). This not only corroborates the insight gained from studies on NSC-34 cells but also demonstrates that the VDAC1-based peptide acts as a neuroprotectant against misfolded SOD1-mediated MN toxicity. Further, these results underline the importance of a direct misfolded SOD1-VDAC1 interaction for the appearance of misfolded SOD1 toxicity. It remains to be shown whether the VDAC1-based (1-20)-N-Ter-Antp peptide also has the same effect on fully mature human motor neurons. Indeed, the results justify more thorough *in vitro* and *in vivo* analysis of the therapeutic potential of the VDAC1-based (1-20)-N-Ter-Antp peptide in treating ALS. These include improving peptide stability, such as by introducing amino acids in the D-conformation and carrying out toxicological studies, as performed with another VDAC1-based peptide specifically inducing cell death of cancer cells (Shteinfer-Kuzmine et al., 2018).

In summary, we have shown here that SOD1 mutants interact with VDAC1 through the VDAC1 N-terminal domain to exert their inhibitory effect on VDAC1 channel conductance. Moreover, we have shown that VDAC1 N-terminal-derived peptides specifically bind mutant SOD1 and inhibit mutant SOD1-induced toxicity in motor neuron-like NSC-34 cells expressing mutant SOD1 or in mouse embryonic stem cell-derived motor neurons. Thus, we suggest that this VDAC1-based peptide represents a new strategy for interfering with mutant SOD1-mediated cell toxicity.

## Author Contributions

AMe, EK, VS-B, and AI designed the research. SA, RG, AS-K, NS, SA-H, YG, JH, and AMa conducted the experiments. SA, RG, AS-K, SA-H, NS, JH, AMa, AMe, EK, VS-B, and AI analyzed the data. VS-B and AI wrote the manuscript.

## Conflict of Interest Statement

The authors declare that the research was conducted in the absence of any commercial or financial relationships that could be construed as a potential conflict of interest.

## Footnotes

1 http://alsod.iop.kcl.ac.uk/als/
